# Impact of spiritual values on tourists’ psychological wellbeing: evidence from China’s Buddhist mountains

**DOI:** 10.3389/fpsyg.2023.1136755

**Published:** 2023-08-03

**Authors:** Ge Zhang, Keji Huang, Shiwei Shen

**Affiliations:** Department of Tourism Management, Ningbo University-University of Angers Joint Institute, Ningbo University, Ningbo, China

**Keywords:** spiritual values, psychological wellbeing, PERMA model, Buddhist mountains, religious tourists

## Abstract

Spiritual values can be a source of meaning for people, and can also determine their feelings, behavior, and mental health. In China’s Buddhist mountains, we collected a total of 400 valid questionnaires from Mount Putuo and Mount Jiuhua, and identified spiritual values as transcendence, general connectedness, inner balance, positive life direction, and special religious feelings. We also explored the impact of these spiritual values on tourists’ psychological wellbeing according to the PERMA model (positive emotion, engagement, relationships, meaning, and achievement). The results revealed that the more easily attained spiritual values (general connectedness, positive life direction, and special religious feelings) had a greater influence on psychological wellbeing than the less easily-attained spiritual values (transcendence and inner balance). Positive emotion and meaning, as components of psychological wellbeing, were strongly influenced by the four spiritual values, whereas engagement, accomplishment, and relationships were influenced by fewer spiritual values. The research contributes to the existing knowledge on spiritual values by analyzing their dimensions and relationships with tourists’ wellbeing from different levels, and also provides empirical suggestions for the sustainable development of religious tourism destinations.

## 1. Introduction

Buddhist mountains are unusual because it blends faith, culture, and the natural environment. Although tourists represent a heterogeneous group (Filep, 2014), they all have values and beliefs. Unlike tourists to other destinations, tourists to the Buddhist mountains experience not only the wonderful natural scenery and charm of the area but also Buddhist culture. Buddhism has the social function of enlightening and purifying the mind. Pilgrimages influence the state of mind and behavioral intention of tourists. A spiritual seeking transition occurs for tourists in the Buddhist mountains. In this process, people undertake a highly personal journey of transformation and hopefully growth, aiming to discover and create their own meaning-generating spiritual orientation. Although the transition can be found throughout the Western modern world, its application in the Chinese context needs to be validated. In particular, the spread of the disease and the uncertainty of COVID-19 challenged people’s mental health (Rathakrishnan et al., 2022). Most people are in fear, stress, and extreme sadness about infection. Many studies have linked psychological factors such as wellbeing to epidemics (Ramkissoon, 2022). And mental health has been linked to spiritual beliefs during the epidemic, suggesting that people may use spirituality for positive mental health (Chirico, 2021). Additionally, the impact of the long-term psychological consequences of coronavirus disease on people’s minds is even more pronounced in the Buddhist mountains (Bella et al., 2021). Natural beauty may produce psychological recovery, promote a sense of wellbeing and improve people’s quality of life (Ramkissoon, 2021). Thus, Buddhism tourism has a positive effect on mental health and the spiritual changes of Buddhist tourists require more research.

Spiritual values play an important role in Buddhist tourists. Destination selection, consumption habits, and other elements of tourist behavior are all influenced by values (Rokeach, 1973; Hofstede, 1980), particularly at Buddhist tourism destinations in China (Huang and Wen, 2021). Values, as ideal states of being that individuals strive toward, serve as standards for judging various actions (Schwartz, 2010). Cultural values can be studied in a range of settings, such as work, education, and tourism. The pursuit of meaning is frequently seen as an aspect of spirituality, and is crucial to pleasure orientation (Dierendonck and Mohan, 2006). As people’s spiritual values are shaped by their social and cultural environment, the East and West have different value systems based on their respective cultural origins. But the range of factors typically used by academics to define and assess values are not entirely applicable to the Buddhist mountains in China. Recently, Huang (2017) has made contributions in this field by identifying five factors that characterize the spiritual values in the context of China’s four great Buddhist mountains. This study similarly focused on China’s Buddhist mountains for the extension. Based on tourist perceptions, it is worth to explore whether spiritual values have different effects on their wellbeing.

The benefits of psychological wellbeing closely resemble the objectives of spiritual values, but this view lacks substantial empirical evidence. Wellbeing is one of the ultimate aims of tourists and lies at the heart of people’s understanding of their psychological state (Seligman, 2011). Wellbeing makes a person’s life meaningful and enjoyable, and people are increasingly looking for ways to improve their wellbeing. Buddhism tourism is often connected with psychological feelings (Huang et al., 2019). People frequently travel to escape their daily routine, alter their moods, and enjoy different attractions. Tourists use their senses to perceive, confront their fears, and feed their souls. Tourism helps people to discover the worth of their existence and the fortitude to persevere in tough situations, thereby improving their overall quality of life. According to scholars, psychological wellbeing (PWB) emphasizes meaning in addition to the wellbeing-related dimensions (Deci and Ryan, 2000). PWB is essentially consistent with Buddhist principles and can bridge the gap between subjective wellbeing theory and religious contexts (Huang et al., 2019). The PERMA model (positive emotion, engagement, relationships, meaning, and achievement) of positive psychology can be used to evaluate how different multicultural environments affect the psychological strength of people or groups (Seligman, 2011). It will be applied in this study to evaluate the impacts of Buddhism tourism on tourists’ psychological wellbeing.

Spiritual values also inevitably affect mental health and wellbeing to a greater or lesser degree. According to self-decision theory (SDT), the achievement of internal goals can enhance wellbeing because it satisfies people’s psychological needs (Deci and Ryan, 2000). Past studies have proved that spirituality values improve people’s wellbeing (Kasser et al., 2014). Although research on various aspects of spiritual values is rapidly growing, the role in psychological wellbeing has been widely under-researched. The search for meaning is the most visible manifestation of the spiritual values that influence PWB. In the context of Buddhism tourism, in addition to simple pleasures, people can also add meaning to their lives through sacred travel (Corvo, 2011). Love, kindness, optimism, and passion have been found to positively enhance subjective wellbeing, but this list does not address meaning. Moreover, subjective wellbeing and psychological wellbeing may differ in facing the same goals (Deci and Ryan, 2000). Spiritual values are more likely to satisfy the essential needs of individuals and thereby enhance psychological wellbeing with an emphasis on self-actualization. Spiritual values tend to guide people’s pursuit of meaning, but the process of pursuing is even accompanied by pain, stress and anxiety. Some studies have shown that seeking meaning is negatively related to subjective wellbeing (Jin et al., 2016). Although viewed as central, the contributions of spiritual values to psychological wellbeing have not been examined adequately (Spännäri and Laceulle, 2021). Consequently, this study aims to reveal the application of spiritual values to the attainment of psychological wellbeing in China’s Buddhist cultural atmosphere. In the following section, we review the literature on spiritual values and PWB in relation to tourism.

## 2. Literature review

### 2.1. Spiritual values

Values are certain states, objects, goals, or behaviors to which people aspire, and which serve as criteria for judging and selecting ways of behaving in specific contexts (Schwartz, 2010). They are idealized rather than currently realized and play a guiding role in people’s search for meaning. Based on the need for socialization, the formation of values is influenced to some degree by one’s social and cultural environment (Schwartz, 1992). Each cultural community has its cultural values. When tourists travel, cultural exchanges occur between them and others. To a certain extent, cultural values can help people to understand their behaviors and feelings (Hsu and Huang, 2016; Huang, 2017). The best examples of religious culture are found at religious locations. It shows that spiritual values can be defined as a particular type and application of cultural values in a religious context. Previously, academic definitions of spiritual values are derived from spirituality. Spirituality is a person’s conscious experience of connecting actual life with noble values (Syihabuddin, 2017). It is an enduring value that directs human life to maintain motivation and force for doing certain activities. Syihabuddin (2017) argues that spirituality is different from religion. The former is the practice of a belief, while the latter emanates from one’s previous beliefs and teachings. Spirituality lacks the institutionalized patterns typical of the various religions, and is rather a personal quest for meaning, purpose, and a sense of connectedness with other beings (Zinnbauer et al., 1997). And then self-transcendence and conservatism in Schwartz’ s values model are identified as spiritual values (Zhang et al., 2014). Some scholars have tended to select relevant concepts from a large range of components, such as convenience, indulgence, leisure, and flaunting (Hsu and Huang, 2016). Recently, spiritual values are summarized by five factors: transcendence, general connectedness, inner balance, positive life direction, and special religious feelings (Huang, 2017). Specifically, individual self-transcendence is the cognition of and belief in a higher form of living beyond the self (Li, 2006). General connectedness refers to the structure of relationships between different individuals, between individuals and society, and between individuals and nature. The term inner balance is often used as a synonym for health, which can be categorized into stressors, personality traits, and health problems (Kenney, 2000). Positive life direction means that one’s life has direction and meaning (Ryff, 2022). The concept of transcendence of the material world and existential nature is established in religion. The special religious feelings have been used to describe dimensions closely related to religion. These five factors well summarize and reflect the definition and role of spiritual values. Furthermore, Huang (2017) have classified the five spiritual value factors into three levels based on their relative importance: Special religious belief and inner balance are at the basic level, positive life direction general connectedness are at the second level, and transcendence is at the highest level. Whether the different levels of spiritual values have different effects needs to be further explored.

While the educational role of spiritual values has been used in many fields, researchers have seldom examined it in tourism (Hannam, 2022). Sites of culture, stately architecture, and scenic beauty attract tourists. They can influence people’s thoughts and behavioral intentions to follow the calling of spirituality (Iliev, 2020). Spiritual values are an important expression of a combination of cultural values and spirituality. The role of spiritual values in mental health has been studied by many scholars. When faced with challenges in life, spiritual values help people in making decisions, managing stress, and overcoming depression (Brown et al., 2013). Additionally, some research has been done to investigate how spirituality assists people in finding their purpose in life, boosting their self-confidence, and providing them with moral support (Rathakrishnan et al., 2022).

### 2.2. Psychological wellbeing

Wellbeing is an important component of healthy living. It includes not only objective factors but also subjective factors such as mental and emotional health (Seligman and Csikszentmihalyi, 2000). In the long term, great effort has been made by researchers to understand the factors that influence wellbeing. The study of positive psychology focuses on the factors that contribute to the pursuit of meaning (Park et al., 2004). Some such pathways have been identified in the field of medical treatment, the workplace, and education. Tourism has been proven to improve tourists’ wellbeing. When people travel, experiencing different locations leads to cultural communications that alter their feelings and mental state (Levin, 2009; Barbara et al., 2020; Mayer et al., 2020). Moreover, much research has explored the relationship between PWB and other factors in the context of scenic spots. Wellbeing may be influenced by individual experiences and pursuits, and scholars have attempted to explain the effects of certain types of journeys on the PWB of tourists.

To analyze the impact of Buddhist culture on tourists, PWB is more applicable than subjective wellbeing. PWB, according to Seligman, is a combination of enjoyment and meaning (Seligman, 2011). Enjoyment comes from pleasurable activities, while meaning comes from a sense of purpose and accomplishment. By understanding the specific components of PWB, we can better apply them to the tourism industry. Seligman (2011) identified five domains: positive emotion, engagement, relationships, meaning, and accomplishment. Positive emotion is an element that refers to pleasure, enthusiasm, comfort, and other similar feelings (Seligman, 2011). It can both reinforce positive emotions and help people to deal with potential negative emotions in the future. Engagement is a state of flow. While people are completely focused on a significant interest during experiential activity, their consciousness of time and other thoughts disappears (Seligman, 2011). As a core domain of wellbeing, relationships refer to connections with others, and positive relationships can be key to achieving and maintaining a healthy state of mind (Noble and McGrath, 2014). Meaning helps to provide purpose in life and the sense of being associated with something stronger than oneself (Kern et al., 2015). It also points to the path to pleasure. Achievement includes satisfaction with what has been achieved and the basic drive to accomplish things (Seligman, 2011).

### 2.3. Spiritual values, psychological wellbeing, and tourism

The coherence between individual values and the environment has an important impact on wellbeing (Sagiv and Schwartz, 2000). An environment that is consistent with the individual’s values facilitates the expression of values and the achievement of goals, while an inconsistent environment hinders it. Values have been widely applied in the tourism environment, and factors that influence the relationship between spiritual values and PWB have often been studied. Hotel trainees, for instance, learn how values and personality factors interact. Considering values allows for a more thorough psychological analysis during tourism research. Moreover, the religious heritage of Buddhist sites helps to increase tourists’ happiness and pleasure. Tourists to Buddhist sites are frequently influenced by the manifestations of Buddhism, the natural environment, and the people they meet, all of which can significantly alter their spiritual values (Huang et al., 2019).

There has been a great deal of research on the relationship between values and wellbeing (Kasser et al., 2014). They involve demographic factors, cognitive and motivational factors, and personality factors of subjective wellbeing (Park et al., 2004). But most studies on the effects have focused on subjective wellbeing. Less attention is currently paid to psychological wellbeing. Spiritual values have psychosocial and religious components to promote mental health (Alborz et al., 2019). Some research revealed relationships between pro-health behaviors, spirituality, and wellbeing (Boswell et al., 2006). Additionally, some researchers have shown a connection between wellbeing and other factors that are somewhat connected to spiritual values, including character strength, personality force, leisure, self-affirmation, self-esteem, and optimism (Matthews and Cook, 2010). All aspects of the character have shown positive correlations with the domains of PWB. Therefore, it is worth exploring whether spiritual values have a similar effect. Tourism and wellbeing are mutually beneficial. Wellbeing is not only promoted after a relaxing journey. Elements of religious tourism include spiritual engagement, learning new things, socializing and feeling a sense of belonging, establishing emotional ties, and finding peace (Bond et al., 2015). However, the question of whether Buddhism affects tourists and promotes PWB requires further consideration and discussion.

In previous research, spiritual values have been strongly associated with wellbeing (Sagiv and Schwartz, 2000), but the impact of different values varies. Most findings indicate that spiritual values are positively related to wellbeing while negatively related to subjective wellbeing (Sagiv and Schwartz, 2000). Therefore, the extent to which spiritual values affect wellbeing needs to be categorized. Many studies have linked the five factors of spiritual values to wellbeing. For example, meaning in psychological wellbeing is contextual, in relation to the personal history and experience (Uwland-Sikkema et al., 2018). Tourism activities make tourists’ positive emotions increase. A fulfilling tourism experience is not only reflected in the acquisition of positive emotions, but also in the meaning of life that tourists gain through tourism (Steger et al., 2006). However, few scholars have explored the classification of different levels of spiritual values. In brief, spiritual values, and PWB, all show correlations at the macro level in Buddhist tourism, and the following theoretical background provides the foundation for our proposed hypotheses.

## 3. Theoretical grounding and hypotheses

### 3.1. Theoretical grounding

Self-decision theory suggests that there are three basic human needs, namely, autonomy, recognition, and relationship need. The satisfaction of these three needs is a key element in achieving wellbeing. Kasser and Ryan (1993) divide its content into external goals and internal goals based on SDT. External goals are primarily closely linked to materialistic values. As internal goals enhance individual wellbeing by satisfying basic human psychological needs, this study focuses on the impact of internal goals (Deci and Ryan, 2000). Healthy values meet an individual’s needs for psychological growth and self-actualization, and such values promote a sense of wellbeing.

Individuals have a greater sense of wellbeing when their values are supported and recognized by the environment, while spiritual values can cause pain when they are not congruent (Sagiv and Schwartz, 2000). What people feel during tourism is not only simple sensory enjoyment, but also a comprehensive experience of spiritual self-realization (Tuo, 2015). Therefore, in the Buddhist mountains, the consistency of the tourist’ s spiritual values with the environment in which he or she lives can have an impact on wellbeing.

### 3.2. Hypothesis formulation

Values moderate the conflict between people’ s life satisfaction in specific areas (Oishi et al., 1999). Huang (2017) divides spiritual values into three levels by the relative importance of factors, the most basic level being special religious belief and inner balance, the second level being positive life direction, general connectedness, and the highest level being transcendence. Different levels of spiritual values may have different impacts. Therefore, the impact needs to be gradually analyzed by starting from different levels of values.

In terms of transcendence, some findings suggest that self-transcendence of spirituality values significantly and positively predicts subjective quality of life (Zhang and Yu, 2014). And a person who identifies with self-transcendent values will have more lasting wellbeing (Bojanowska and Piotrowski, 2018). Transcendence has been more frequently studied in association with the PERMA, especially from a self-transcendence perspective.

Individuals with self-transcendence show higher levels of self-esteem and produce more positive affective experiences related to love (Canevello and Crocker, 2015). And self-transcendent significantly predicted reductions in anxiety, depression, loneliness (Liu et al., 2021), and the promotion of a peaceful mind (Canevello and Crocker, 2015). Transcendence provides an environment where people can dig deep inside themselves, free from social and physical constraints (Schwartz, 1992). As a result, people are willing to engage at that location. Individuals with self-transcendent values have less defensive responses to self-threatening information, experience more social relationship-oriented emotions, and inspire more pro-social behaviors that lead to lasting wellbeing (Kang et al., 2018; Liu et al., 2022).

Among the studies related to transcendence, relationships have been widely focused on by scholars. Friendship and ecumenism express the concern for the interests of others. Individuals with a self-transcendence orientation may provide more encouragement and support to their partners (Feeney et al., 2013). Being sincere and showing behavior that is consistent with inner feelings, attitudes and beliefs helps individuals develop positive interpersonal relationships (Bojanowska and Piotrowski, 2018). Individuals combine their own interests with the interests of the group, pay more attention to the happiness and needs of others, and make themselves feel happy while benefiting others. Individuals are usually able to understand, appreciate, and tolerate others (Schwartz, 1992). Transcendent values contribute to harmonious interpersonal relationships (Crocker et al., 2016), bringing more connection and satisfaction. By transcending interests and desires, people can also be harmoniously connected to others and society. Self-transcendence values lead to positive interpersonal relationships and increased social support, contributing to a lasting sense of wellbeing (Crocker et al., 2016). At the same time, people’s perspectives on gain and loss are affected, and there is a willingness to forgive others. Individuals focus on personal issues, and their position in the group, and seek social prestige, and achievement.

Referring to the PWB model, a series of related psychological and situational dimensions are required for self-transcendence to occur (Neuhofer et al., 2020). For example, gratitude, a character strength belonging to the transcendental virtues, is positively correlated with all dimensions of PWB (Butler and Kern, 2016). Transcendental virtues have been found to predict the five dimensions of PWB, and are crucial to the quality of one’s spiritual world. Therefore, the following hypotheses are proposed:

*H1a–e*: Transcendence positively affects tourists’ positive emotion, engagement, relationships, meaning, and accomplishment.

Connectedness may be unavoidable, not only from human to human, but also between people and the environment (Hofstede, 1980). Good relationships are a common factor in various methods of predicting wellbeing. General connectedness includes not only relationships with others, but also relationships in society as well as the natural environment. Relationships are a basic need, and when they are satisfied, individuals can not only relieve their stress, but also feel happier (Canevello and Crocker, 2017). People with positive relationships are happier and healthier than wealth and fame. The values of self-growth and interpersonal connectedness promote lasting wellbeing (Deci and Ryan, 2000).

In addition, wellbeing may be enhanced by various social interactions, especially leisure activities (Berdychevsky et al., 2013; Ramkissoon, 2020). Generally speaking, interactions in tourism are an important source of wellbeing (Zhang et al., 2020). Several studies have proposed that positive host-guest interactions can release wellbeing (Chen et al., 2017). And physical distances directly affect the feelings between tourists and hosts, while the way and degree of social interaction affect wellbeing (Kim et al., 2020). Most of the studies on tourist wellbeing show that the gaining of positive emotions by tourists is due to the role of interaction (Nawijn, 2011). Many studies consistently show that positive emotions are associated with an easy-to-socialize personality and that outgoingness enhances the feeling of wellbeing (Eysenck and Jamison, 2010). The effect of social relationships on subjective wellbeing was more significant for introverted individuals.

Pro-social behaviors enhance social integration and contribute to the establishment and maintenance of interpersonal relationships, thereby increasing individuals’ lasting experiences of wellbeing. On the one hand, socially engaging emotions enable interpersonal affective connections (Tamir et al., 2015). On the other hand, pro-social behaviors enhance interpersonal integration (Aknin and Human, 2015). The stability of social relationships enhances emotional stability. And research has shown a positive association between emotional stability and wellbeing (Bajaj et al., 2019). Individuals develop a sense of harmony, including with themselves, with others or with society, and with the world. Accordingly, we propose the following hypotheses.

*H2a–e*: General connectedness positively affects tourists’ positive emotion, engagement, relationships, meaning, and accomplishment.

Inner balance is an ideal state people seek. In the yin–yang dialectic, the concept of balance and harmony is captured in the theory of mature happiness (Wong, 2016). The concept of happiness emphasizes inner harmony. A cross-cultural study found that people often define inner balance in terms of the inner cosmos and personal relationships. People combine internal and external resource es to seek a balanced life in which they live in peace with themselves, others, and the world (Verme, 2009; Wong, 2014). Qualities such as self-acceptance are considered to be related to happiness and joy. Whether it is pleasure or pain, individuals can usually rely on their inner strength to cope with what they experience, and maintain lasting wellbeing. And it can manifest as a state of constant harmony, and calm. Agreeableness plays an instrumental role in wellbeing, which is enhanced by creating environments and life events that make people happy (McCrae and Costa, 1999). The internal dimension can increase wellbeing and quality of life. We thus propose the following hypotheses.

*H3a–e*: Inner balance positively affects tourists’ positive emotion, engagement, relationships, meaning, and accomplishment.

Positive life direction is similar to self-development (Rokeach, 1973). The search for meaning is a constant theme in our lives, and a positive life direction gives people meaning and purpose to maintain social functioning and stability. A positive evaluation of the meaning of life is an important source of happiness. Individual values determine the setting of goals and motivate the pursuit of them (Zhang et al., 2014). Wellbeing is related to some cognitive factors such as self-esteem, optimism, and life goals (Hills and Argyle, 2001). The role of specific goals on personal wellbeing is closely related to the interaction between the various values or goals. Specifically, a conflict between values and goals will reduce wellbeing. When a value goal conflicts with a need, it can produce psychological problems such as anxiety and depression (Mineka and Zinbarg, 2006). The goal theory of subjective wellbeing suggests that goals and values determine a person’s wellbeing, and that factors such as goal attainment and life satisfaction (Diener et al., 1999). Steger’s approach which distinguishes among purpose, significance, and coherence defines the needs for meaning: purpose, efficacy, moral worth, and self-worth (Martela and Steger, 2016). This means that goals are associated with meaning. It is important to note that positive life direction may have a positive impact on wellbeing. For example, the hope is positively associated with partners’ wellbeing (Botha et al., 2022). People who are good at giving positive meaning to events have higher levels of wellbeing in ordinary life time.

Positive life goals tend to be more focused on intrinsically valuable goals. Studies have shown that individuals who focus on internal value goals (e.g., self-growth, emotional closeness) have higher life satisfaction, and exhibit fewer health problems such as depression and anxiety than external goals (e.g., possessions, social prestige) (Kasser and Ryan, 1993). The internal goals, as a reflection of values originating from spirituality, facilitate the integration of goal systems that result in an optimal mental health, including greater subjective wellbeing, and adaptability (Sheldon and Kasser, 1995). In the field of tourism, scholars such as Berdychevsky et al. (2013) concluded that tourism activities bring positive emotions to women. And the sense of wellbeing can be enhanced when tourists travel. Typically, this requires clear goals and immediate feedback. Because tourism activities are highly experiential, the following hypotheses are proposed.

*H4a–e*: Positive life direction positively affects tourists’ positive emotion, engagement, relationships, meaning, and accomplishment.

The terms “religion” and “spirituality” are ambiguous and frequently used interchangeably. Spirituality develops more around the idea of transcendence as opposed to religion, which frequently relates to ideas, actions, rituals, and ceremonies connected to an established tradition. Spirituality is not limited to religious beliefs (Chirico et al., 2019). Despite having some differences, they are all related to wellbeing. Religions can serve as an influential framework for meaning and construction in relation to beliefs, expectations, and goals since they most frequently directly address problems pertaining to the sacred and the transcendent (Lewis Hall and Hill, 2019). Patients with religious beliefs can achieve spiritual wellbeing by praying to God and finding a connection to the sage, thereby gaining comfort and increasing their willingness to actively seek treatment (Peteet and Balboni, 2013). Individual prayers may attain gratitude, humility, forgiveness, and so on.

Additionally, spirituality involves a high level of cognitive processes, and integrates the entrepreneur’s moral, social and religious values (Kolany, 2010). Certain character strengths, especially spirituality, tend to enhance wellbeing. Spirituality gives believers a sense of the sacred and brings them closer to their faith. Equally, spirituality has been shown to predict the components of wellbeing. Correlational studies have been carried out that spirituality is related to different variables such as positive mental health, wellbeing, and hope (McEntee et al., 2013). Spirituality helps people relax. There are a variety of religious and non-religious forms in older age groups, all of which can be relevant factors in meaning-seeking (Spännäri and Laceulle, 2021). The social and communal settings of religion and spirituality remain profoundly important in meaning pursuit. And spirituality was presented as a positive mediator between psychological resilience and success (Margaça et al., 2022). A study shows that beliefs and life goals can help to develop wellbeing (Cotton et al., 2009). Therefore, the following hypotheses are proposed.

*H5a–e*: Special religious feelings positively affect tourists’ positive emotion, engagement, relationships, meaning, and accomplishment.

## 4. Methodology

### 4.1. Research sample

This study considers Mount Putuo and Mount Jiuhua, two of the four great Buddhist mountains of China, which are located in the provinces of Zhejiang and Anhui, respectively, in eastern China. Both have a developed economy and convenient transportation. Mount Putuo attracts a steady stream of tourists because it is dedicated to Avalokitesvara, the Goddess of Mercy who embodies compassion and wisdom in the hearts of followers. Mount Putuo attracted more than 6 million pilgrims in 2020, generating nearly US$7.4 billion (Putuoshan Government, 2021). Similarly, Mount Jiuhua is the site of the dojo of Ksitigarbha. Many pilgrims visit here on special occasions to seek peace and make wishes. People visit the Buddhist mountains not only to realize their aspirations for a better life, but also to appreciate nature, the magnificent temple architecture, and local customs. And Mount Jiuhua received more than 7.9 million visitors and generates more than US$11.4 billion in revenue during the year 2020 (Anhui Bureau of Statistics, 2021). These Buddhist mountains in China are the focus of this study because they constitute an important and heavily visited subset of religious sites. As a result, surveying tourists at both sites can help to better understand their spiritual values and PWB.

### 4.2. Measures

A three-part questionnaire was prepared. The first part measured spiritual values through the five spiritual values identified by Huang, namely transcendence, general connectedness, inner balance, positive life direction, and special religious feelings (Huang, 2017). Based on China’s Buddhist mountains, we made some modifications to the items in Huang (2017) study to increase the accuracy of the scale test. For example, “This trip gives me a sense of belonging,” “This trip makes me feel that having good relationships with others is important,” “This trip gives me a different sense of space and time,” “This trip makes me This trip makes me feel a part of the community in which I live.” There were 34 items in this section.

The second part of the questionnaire was comprised of five domains to measure PWB, adapted from Butler and Kern, namely positive emotion, engagement, relationships, meaning, and accomplishment (Butler and Kern, 2016). With 15 questions (three per PERMA domain), we added a question to each of the five domains of the PERMA model on the basis of Butler and Kern’ s questionnaire in order to improve the scale test’s accuracy. Thus, there were 20 items in this scale.

The questions in the first two parts of the questionnaire were answered using a 7-point Likert scale, where 1 = “strongly disagree,” 2 = “disagree,” 3 = “slightly disagree,” 4 = “neutral,” 5 = “slightly agree,” 6 = “agree,” and 7 = “strongly agree.” The third section collected demographic information to better understand the tourists’ characteristics, including their gender, age, occupation, education level, religion, and visit frequency.

SPSS and AMOS software were used for the data analysis. First, exploratory factor analysis and reliability analysis of the first section were conducted. Questions with loading coefficients on their factors lower than 0.5 were deleted (Ferguson and Cox, 1993). The remaining 21 questions were grouped into five dimensions based on the factor loadings after rotation. Following factor validation analysis, the correlation between spiritual values and PWB was analyzed to verify whether the hypotheses were valid.

### 4.3. Data collection

The data were collected in 2021 from 21 to 26 June at Mount Jiuhua and from 9 to 16 July at Mount Putuo. In the formal data collection procedure, the researchers distributed 471 questionnaires; 400 were valid, with a valid response rate of 84.9%, with 71 invalid questionnaires including those unfinished, ambiguous, and all questions were rated the same. All of the participants were tourists at Mount Jiuhua and Mount Putuo. Table 1 shows the demographic information: 207 (51.7%) of the respondents were male, 182 (45.5%) were married, and 198 were single (49.5%). The largest age cohort (48.5%) was 19–30 years of age. Most respondents had a tertiary education: 27.9% had a junior college education and 235 (58.7%) had an undergraduate education. The respondents came from all walks of life, and showed a relatively average distribution. Most were working for companies, but private businesses and students also accounted for a large proportion. Interestingly, 201 (50.2%) of the respondents were neutral in their religious beliefs, while 165 had a deep faith. Nearly 36% of them visited religious sites three to five times per year. Most of the tourists came from Zhejiang, Fujian, Anhui, and Jiangsu provinces, while some were from inland provinces such as Shandong and Henan.

**Table 1:** Demographic information table.

Demographic	Items	Total (*N* = 400)	
		*n*	%
Gender	Male	207	51.7
	Female	193	48.2
Marital status	Married	182	45.5
	Single	198	49.5
	Not specified	20	5.0
Age (years)	18 or younger	32	8.0
	19–30	194	48.5
	31–40	119	29.7
	41–50	44	11.0
	Over 50	11	2.8
Education	High school or lower	29	8.2
	Junior college	114	27.9
	Undergraduate	235	58.7
	Graduate or above	22	5.5
Occupation	Government department	34	8.5
	Company	122	30.5
	Private business	79	19.7
	Freelance work	49	12.2
	Student	69	17.2
	Other	47	11.7
Buddhism belief	Very weak	34	8.5
	Neutral	201	50.2
	Very strong	165	41.2
Annual frequency of religious travel	Fewer than 3 times	129	32.2
	3–5 times	144	36.0
	6–9 times	59	14.7
	More than 10 times	68	17.0

## 5. Results

### 5.1. Measurement model

#### 5.1.1. Multicollinearity and normality

We used SPSS 26.0 and AMOS 22.0 to analyze the questionnaire data. The first step in the analysis was Harman’s single-factor test to check for common method variance (CMV). The number of factors was set at 1 and 49.978% of the total variation was explained by the first common component, which was less than the empirical criterion of 50% (Podsakoff et al., 2012). According to Guide and Ketokivi (2015), the use of correlation and single-factor tests is no longer considered acceptable in research. Therefore, this study employed an unmeasured latent method construct (ULMC) technique as suggested by Podsakoff et al. (2012). The fit indices changed little from the original model, with the RMSEA changing from 0.044 to 0.041, a change of no more than 0.05. The change in SRMR was also less than 0.05. And the CFI and TLI both changed by less than 0.1. This implied that there was no serious common method variance in the measurements. The data distribution showed an absolute value of less than 3 and the skewness values for all items varied from −1.198 to −0.247. The absolute value of kurtosis for all elements ranged from −0.494 to 1.798, well below the cut-off of 8. Thus, the data followed the normal distribution.

#### 5.1.2. Reliability analysis and exploratory factor analysis of spiritual values

The Cronbach’s alpha for the overall model was 0.974, indicating that the data were real and valid. We conducted exploratory factor analysis (EFA) for the scale on spiritual values (Table 2). We modified the questions to fit the sites of China’s Buddhist mountains. Similar to the results of Huang (2017), the questions with loading coefficients lower than 0.5 (Smith, 2012) were excluded, namely SV4, SV5, SV10, SV11, SV13, SV14, SV15, SV16, SV17, SV22, SV23, SV24, and SV34. The remaining 21 items formed five dimensions using the rotary factor predominantly. These were transcendence, general connectedness, inner balance, positive life direction, and special religious feelings.

**Table 2:** EFA results for spiritual values (*N* = 400).

Items	Latent variables					AVE	α
	1	2	3	4	5		
SV1	0.785					0.611	0.820
SV2	0.747						
SV3	0.741						
SV6		0.775				0.563	0.865
SV7		0.767					
SV8		0.653					
SV9		0.628					
SV12		0.581					
SV18			0.591			0.604	0.854
SV19			0.717				
SV20			0.753				
SV21			0.789				
SV25				0.626		0.615	0.916
SV26				0.659			
SV27				0.707			
SV28				0.756			
SV29				0.732			
SV30				0.601			
SV31				0.631			
SV32					0.806	0.737	0.847
SV33					0.781		

SV, spiritual values of tourists; AVE, average variance explained; α, Cronbach’s alpha.

#### 5.1.3. Convergent validity

As shown in Table 3, the confirmatory factor analysis (CFA) results showed that the spiritual values scale was an acceptable model. Composite reliability (CR) values reflect the extent to which construct indicators indicate potential, ranging from 0.824 to 0.918, above the recommended value of 0.7. The resulting data were within the acceptable range for both reliability and validity (CMIN/DF = 1.762, CFI = 0.957, GFI = 0.862, IFI = 0.957, TLI = 0.952, NFI = 0.905, RMSEA = 0.044).

**Table 3:** CFA results for spiritual values (*N* = 400).

Constructs and indicators	FL	SMC	CR
Transcendence (T)			0.824
SV1: This trip makes me see things from new and different points of view.	0.773	0.598	
SV2: This trip gives me a sense of belonging.	0.847	0.717	
SV3: This trip makes me forgive people who have wronged me in the past.	0.719	0.517	
General connectedness (GC)			0.865
SV6: This trip makes me feel that having good relationships with others is important.	0.761	0.579	
SV7: This trip makes me feel close to nature.	0.756	0.571	
SV8: This trip makes me experience moments of peace during a devastating event.	0.671	0.450	
SV9: This trip makes me feel connected to other people.	0.779	0.607	
SV12: This trip makes me enjoy being of service to others.	0.780	0.609	
Inner balance (IB)			0.858
SV18: This trip gives me a different sense of space and time.	0.697	0.486	
SV19: This trip makes me feel good about myself.	0.756	0.572	
SV20: This trip gives me a sense of balance in my life.	0.848	0718	
SV21: This trip makes me feel fulfilled in life.	0.799	0.639	
Positive life direction (PLD)			0.918
SV25: This trip gives my life meaning and purpose.	0.809	0.655	
SV26: This trip enables me to receive emotional support from my relationships.	0.744	0.554	
SV27: This trip helps me find inner resources to deal with uncertainty in life.	0.845	0.713	
SV28: This trip makes me value my life.	0.823	0.678	
SV29: This trip makes me feel that a loving connection exists between all optimistic people.	0.800	0.641	
SV30: This trip helps me discover my strength in times of struggle.	0.753	0.567	
SV31: This trip makes me feel a part of the community in which I live.	0.705	0.497	
Special religious feelings (SRF)			0.849
SV32: This trip makes me feel connected to the Buddha or another higher power.	0.839	0.703	
SV33: This trip makes me feel that inner strength is related to a belief in the Buddha.	0.878	0.771	

FL, factor loading; SMC, squared multiple correlations; CR, composite reliability.

Additionally, for the psychological wellbeing (PWB) scale, the results of factor loading (FL), CR, and squared multiple correlations (SMC) data were shown in Table 4. All five dimensions had CR values greater than 0.8. The average variance extracted (AVE) figures indicated good validity with values between 0.665 and 0.772 (greater than 0.5).

**Table 4:** CFA results for PWB (*N* = 400).

Constructs and indicators	FL	SMC	AVE	CR	α
Positive emotion			0.772	0.931	0.931
P1: I am passionate about life.	0.895	0.801			
P2: I am joyful about life.	0.886	0.784			
P3: I am positive about life.	0.876	0.768			
P4: I am content with life.	0.857	0.735			
Engagement			0.665	0.888	0.887
E1: I often go on trips.	0.762	0.581			
E2: I feel excited and interested when on a trip.	0.871	0.759			
E3: I often lose track of time when on a trip.	0.776	0.602			
E4: I often become absorbed when on a trip.	0.848	0.719			
Relationships			0.657	0.884	0.880
R1: Neighbors are willing to help me.	0.723	0.523			
R2: Government workers are willing to help me.	0.735	0.540			
R3: I feel loved in my life.	0.886	0.785			
R4: I feel satisfied with my relationships.	0.883	0.780			
Meaning			0.735	0.917	0.917
M1: I have a clear sense of direction in my life.	0.855	0.731			
M2: I feel worthwhile when I am on a trip.	0.853	0.728			
M3: I know how to live my life.	0.874	0.764			
M4: I feel that my life is valuable and worthwhile.	0.848	0.719			
Accomplishment			0.695	0.901	0.901
A1: Going on trips helps me to grow and develop.	0.831	0.690			
A2: I have achieved important goals.	0.823	0.677			
A3: I have performed the duties of my job.	0.846	0.715			
A4: I have done well during my travel.	0.835	0.698			

#### 5.1.4. Discriminant validity

For the spiritual values scale, we tested for discriminant validity. As shown in Table 5, most of the square root values of AVE on the diagonal were greater than the other values, which is an acceptable range. Therefore, the spiritual values scale has good discriminant validity.

**Table 5:** Discriminant validity results for spiritual values (*N* = 400).

Variable	SRF	PLD	IB	GC	T
SRF	0.858				
PLD	0.682	0.784			
IB	0.521	0.808	0.777		
GC	0.618	0.8	0.716	0.75	
T	0.666	0.666	0.648	0.649	0.782

SRF, special religious feelings; PLD, positive life direction; IB, inner balance; GC, general connectedness; T, transcendence.

Next, the results were confirmed by the discriminant validity of the PWB. Table 6 shows that for most variables, the square root of the average variance explained (AVE) was greater than the composite reliability (CR) score confirming the differentiation of the five antecedent variables. Most discriminant validity results were satisfactory, including those for meaning, positive emotion, special religious feelings, inner balance, general connectedness, and transcendence. Therefore, the model had good validity.

**Table 6:** Discriminant validity results for PWB (*N* = 400).

Variable	A	M	R	E	P
A	0.834				
M	0.851	0.857			
R	0.778	0.820	0.811		
E	0.812	0.808	0.826	0.815	
P	0.769	0.837	0.751	0.843	0.879

A, accomplishment; M, meaning; R, relationships; E, engagement; P, positive emotion.

### 5.2. Structural model

Testing the related indexes of structural equation modeling was important to establish whether the assumptions were tenable. The overall model results were good (CMIN/DF = 2.995, IFI = 0.885, CFI = 0.884, TLI = 0.873, NFI = 0.837, RMSEA = 0.07), and met the criteria for further analysis.

### 5.3. Hypothesis testing

**Figure 1:**
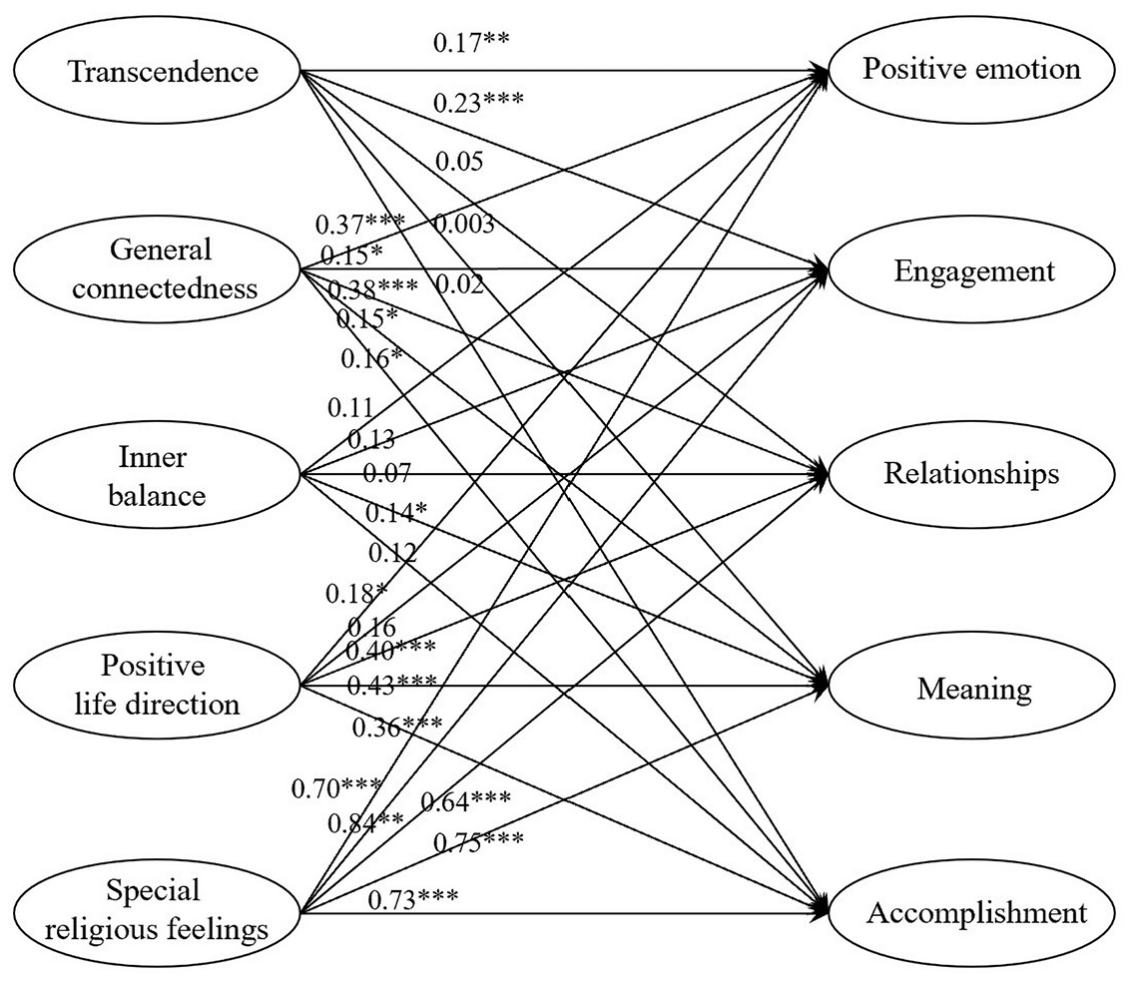
The influence of spiritual values on psychological wellbeing. * *p *< 0.05, ** *p* < 0.01, *** *p* < 0.001.

As shown in Figure 1, the coefficients for the effect of transcendence on positive emotion and engagement were 0.17 (*p* < 0.01) and 0.23 (*p* < 0.001), respectively, supporting H1a and H1b, but H1c–e were not supported. General connectedness positively affected tourists’ positive emotion, engagement, relationships, meaning, and accomplishment (PERMA), supporting H2a–e. According to the model in Figure 1, the coefficient for the effect of inner balance on meaning was 0.14 (*p* < 0.05), supporting H3d. However, inner balance had no significant effect on positive emotion, engagement, relationships, or accomplishment. Positive life direction had a significant effect on positive emotion, relationships, meaning, and accomplishment, but no significant effect on engagement. Therefore, H4a–e were partially supported. Special religious feelings had a significant positive effect on all aspects of PERMA, supporting H5a–e.

## 6. Discussion

The present study was designed to determine the effect of tourists’ spiritual values on PWB at Buddhist sites in China. The results produced four key findings. First, the more achievable the spiritual values, the greater their impact on PWB. In most cases, the five dimensions of PWB were positively related to general connectedness, positive life direction, and special religious feelings, as both ordinary tourists and pilgrims were found to share these spiritual values. People were able to interact with other people, nature, and other aspects of their lives while traveling and find a positive direction for their lives. At the same time, in the special religious setting of the Buddhist mountains, people were inevitably influenced by the cultural atmosphere. Thus, they had a sense of awe, experienced special religious feelings, and actively regulated their behavior, which is a distinctly different scenario from regular tourism.

Second, the more difficult the spiritual values were to attain, the less they affected PWB. Only one or two factors of PWB were significantly influenced by transcendence and inner balance, because the pursuit of higher perceptions and the achievement of ideological growth is accomplished by very few people. Inner balance requires achieving a state of equilibrium with both the external environment and the inner mental state. A few people are influenced by others and their environment to experience transformation in a blinding flash and leave their past life to better influence others. However, these instances are rare and do not lead to a general increase in the level of PWB.

Third, the more unique components of PWB, positive emotion and meaning, were significantly influenced by spiritual values. PWB was significantly affected by four of the spiritual values. Positive emotion and meaning can be enhanced by feeling deeply. Spiritual values are deeper conceptions that influence subsequent behavior and thoughts, resulting in a greater level of wellbeing rather than just a passing influence. When values are profoundly affected, PWB varies substantially.

Fourth, engagement, relationships, and accomplishment were significantly influenced by fewer antecedent variables. They were all impacted by three of the spiritual values and were not representative. Engagement was affected by transcendence, general connectedness and special religious feelings. Relationships and accomplishment were both impacted by general connectedness, positive life direction and special feelings. They have also been recognized in ordinary travel settings and, as a result, were less affected by more profound factors such as spiritual values. The factors most obviously connected with wellbeing had a greater influence. Next, we discuss the relationship between the five factors used to define spiritual values and the five dimensions of PWB separately.

### 6.1. Transcendence

Transcendence had a significant impact on positive emotions and engagement. By knowing oneself, transcendence helps people to consolidate positive emotions and thus better cope with future hardships. Research has found that transcendence through the power of faith increases positive emotions and decreases negative ones, and in this way promotes happiness (Canevello and Crocker, 2015; Liu et al., 2021). As with previous studies, self-transcendence leads to more lasting feelings of wellbeing (Bojanowska and Piotrowski, 2018). Similarly, Buddhist sites not only help individuals to self-affirm, maintain an optimistic attitude, and promote self-confidence to meet their challenges, but also help people to clarify their goals in life, including the goal of transcendence (Cohen and Sherman, 2014). The findings of Kang also mentioned that transcendence causes people to experience more emotions and evoke more pro-social behaviors that lead to the experience of wellbeing (Kang et al., 2018). On the other hand, transcendence also enhances engagement because it motivates people to actively try to overcome the difficulty no matter how hard it seems, so as to achieve different leapfrog development. Engagement means exploring, seeking a sense of belonging, and pursuing knowledge. It depends on positive mental traits including love and spirituality (believing in a higher purpose and the importance of personal development) (Park et al., 2004). During the journey, different social relationships can inspire a love for individuals, and Buddhism implies a spiritual dimension. Self-transcendence practice activities stimulate people’s adaptive potential (Cohen and Sherman, 2014). In the process, problem-solving allows them to further affirm themselves and promote deep engagement, while reaching the final goal enhances their wellbeing. Transcendence is not only an affirmation of oneself but also implies an understanding of the motives, actions, and feelings of others. Engagement in Buddhist tourism enhances people’s sense of meaning from positive events in their lives (Kang et al., 2018). Here people appreciate the beauty and engage deeply with Buddhist culture. These all become important sources of meaning in their lives.

### 6.2. General connectedness

General connectedness promotes the accomplishment of highly interdependent goals and increases wellbeing by enhancing interpersonal cooperation (Cameron and Dutton, 2003). When the basic needs of relationships are met, individuals can relieve themselves of stress and feel happier (Canevello and Crocker, 2017). Various studies with different samples have found that love, kindness, and teamwork are the most important predictors of positive relationships (Canevello and Crocker, 2017). These three character strengths can be conducive to starting and maintaining relationships. Briefly, exposing people to hope and general connectedness to themselves, others, and an environment enhances these character strengths (Tamir et al., 2015). As a basic human need, connectedness is a key indicator of happiness, as it involves being valued by others and having satisfying relationships (Peterson et al., 2007; Seligman, 2011). Having close friends, family, and social support groups helps individuals to build physical, mental, and psychological resources (Perissinotto et al., 2012). In addition, people’s level of wellbeing also depends on those with whom they associate. Spiritual values seem to play a key role in maintaining interpersonal relationships (Eysenck and Jamison, 2010). Those with high emotional intelligence can understand their own and others’ feelings and motivations and maintain positive interpersonal relationships. Significantly, one thing that cannot be overlooked in achievement is social support, which comes from general relationships between people. Other people provide different perspectives that reflect on themselves. This lays a foundation for continuous improvement. During travel, tourists must live with others in different ways than before. This contributes to the value of relationships. Previous studies have pointed out that interactions in tourism are an important source of wellbeing and that positive host-guest interactions can bring the pleasure of tourism (Zhang et al., 2020; Ramkissoon, 2022). Thus, social support obtained through general connectedness can play a part in these situations. In addition to relationships with people, the physical and natural environment can also influence psychological states and mental health (Deci and Ryan, 2000). As studies have pointed out, people enjoy the health and happiness that comes from being connected to the natural environment (Ramkissoon, 2021). In the Buddhist mountains, being surrounded by sacred natural and built environments triggers feelings of awe (Huang et al., 2019). Tourists feel closer to the Buddha and Bodhisattvas here, while also being influenced by the monks and believers. Most people unconsciously regulate their behavior under these conditions and become more willing to do charitable deeds and participate deeply in ritual activities. Even after leaving the holy place, people feel the meaning of their existence and a sense of happiness because of the purification of their bodies and mind.

### 6.3. Inner balance

Inner balance means being healthy in both body and mind. People find their true selves, reject the bad, and accept the positive. In fact, balance means adjusting oneself and analyzing and solving problems. A common value in China is to seek the balance between yin and yang (Kenney, 2000). Regardless of the external environment, organisms can maintain relative stability in their internal environment (Wong, 2014). This ability is of key importance to the survival of the individual. It is a control system that achieves dynamic balance by regulating our behavior and consciousness. Promoting affective attitudes toward place can lead to psychological recovery, and the health that comes from natural connections can contribute to a sense of wellbeing (Ramkissoon, 2021). Inner balance also emphasizes the balance of the mind, which successively supports the balance of the body. This requires clearing the mind, putting all things aside, living in the present moment, and understanding the nature of life. Harmony with self and harmony with society is the essential characteristic of health and happiness (Wong, 2016; Yang and Xue, 2016). Thus, inner balance provides a deeper sense of wellbeing than simply triggering superficial positive emotions, engaging people in activities, maintaining relationships with others, and achieving more.

### 6.4. Positive life direction helps

Positive life direction helps in the pursuit of wellbeing. Realizing one’s self-worth, pursuing goals, internal inspiration, and personal fulfillment all increase the sense of wellbeing (Botha et al., 2022). Differences in goals can affect wellbeing, and positive life direction is what motivates tourists to travel. According to the SDT, the realization of internal goals can satisfy people’s basic psychological needs and improve wellbeing (Deci and Ryan, 2000). Thus, when people are motivated by attractions, they are motivated by the pursuit of positive goals and the desire to gain something from their trip. Positive life direction is closely related to the elements of PWB (Wagner et al., 2020). Some character strengths such as hope and optimism seem to play a key role (Lyubomirsky and Layous, 2013). Hope can help people to set goals and reduce negative thoughts. Optimism refers to the positive anticipation of future events (Carver et al., 2010). It is also a component of positive life direction and has an important role in people’s adaptation to different environments (Huppert and So, 2013). Longitudinal studies have found a positive correlation between optimism and wellbeing (Huppert and So, 2013). Individuals gain greater satisfaction when they pursue goals that are recognized by others. Specifically, if people’s behavior is consistent with the values of others and can be supported and encouraged, this positive feedback increases their wellbeing (Oishi and Sullivan, 2010). Thus, a positive life direction allows people to act with optimism and maintain positive emotions about future events. When people feel they have value and meaning, their overall wellbeing is enhanced.

### 6.5. Special religious feelings

Special religious feelings can give confused people strength and improve their mental health (Brown et al., 2013). By relying on the power of the hyperphysical, religion can psychologically regulate individuals and smoothly the psychological and emotional damage they have suffered. Religious beliefs can lead to spiritual wellbeing through prayer and the search for a connection with the supreme being (Peteet and Balboni, 2013). Engaging in pro-social behaviors may result in positive effects on psychological wellbeing (Ramkissoon, 2020). At the same time, social connection and a sense of belonging are necessary components of psychological wellbeing. All of these can be achieved through religious activities and rituals. To connect with God or Buddha and feel supported, many people travel great distances to visit Buddhist sites, engage with other believers, and worship devoutly (Peteet and Balboni, 2013). In addition, through contact with various Buddhist symbols, such as traditional temple architecture, cultural rituals, and monks, tourists become more willing to follow the positive direction advocated by Buddhist culture (Huang et al., 2019). Curiosity, social competence, appreciation of beauty, gratitude, and spirituality have all been associated with meaning (Kleiman et al., 2013). For most people, curiosity may motivate a search for meaning in life. Spirituality, appreciation for beauty and excellence may all be important sources of meaning. The search for meaning may also promote the formation of close social relationships. Several factors linked with religion can be relevant to meaning (Spännäri and Laceulle, 2021). During pilgrimages, people share their beliefs and express admiration, devotion, and worship. Thus, pilgrimages seem to promote mental health by improving spirituality (Papazoglou et al., 2021), which can increase wellbeing. Faith and purpose in life contribute to the development of psychological wellbeing, which may be an important characteristic to cultivate.

## 7. Conclusion and implications

This study explored how the spiritual values of tourists affect their PWB when they visit the Buddhist mountains in China. Transcendence, general connectedness, inner balance, positive life direction, and special religious feelings were all found to be factors of tourists’ spiritual values. Overall, transcendence and inner balance, the more difficult spiritual values to attain, had less impact on the five PERMA elements of PWB than the other three more attainable spiritual factors. The first two dimensions of PWB, positive emotion and meaning, were more significantly affected by spiritual values than the remaining three factors, namely engagement, relationships, and accomplishment.

### 7.1. Theoretical contributions

First, our findings provide strong support for the use of spiritual values and PWB measures at Chinese Buddhist sites. The questionnaire results substantially supported the applicability of the two scales in this setting. Theoretical contributions are made by exploring the impact of spiritual values on tourists’ psychological wellbeing supported by the theory of SDT. This study confirmed the findings connected to spiritual values and PWB theories. This suggests the benefits of constructing a positive psychology framework system.

Second, research in the field of tourism has shown the relevance of spiritual values and PWB theory. However, these theories have been underutilized in tourism research, and the results of this study allow for better use of them. Furthermore, the association between the two has not previously been investigated. Several dimensions, such as special religious feelings, were unexpectedly dominant in predicting PWB. This draws attention to the importance of religious feelings in other related studies.

Third, our findings suggest the importance of studying spiritual values and PWB in the context of tourism in China’s Buddhist mountains. Spiritual values can also promote PWB among tourists. Specific Buddhist activities that promote wellbeing can thus have a positive impact on the overall evaluation of a trip. These findings can be used by psychologists in work or clinical settings in addition to their contribution to the field of positive psychology. By designing and implementing interventions to help individuals reshape their spiritual values during visits to religious attractions, psychologists could help people to apply them in their daily lives and thus achieve higher levels of wellbeing and its various components.

### 7.2. Practical implications

First, our findings highlight the importance of spiritual values in improving wellbeing during religious tourism. The aim is to improve people’s mental health, especially in the context of the COVID-19 pandemic. Our study expands the concept of spiritual values to include a notion of holistic harmony that integrates values, culture, and spirituality. The results can help tourists to deepen their inner being rather than focus solely on the external environment. The long-term goal of human beings is to find balance among the various aspects of their own lives and environments. Additionally, managers could use correlations to assess the atmosphere in a given setting and determine which tourists may be at risk for psychological distress or spiritual problems.

Second, it is also crucial for tourism managers to include a broad awareness of tourists’ spiritual values and PWB at the planning stage. By promoting activities and contacts, research findings can enhance multiple aspects of tourists’ wellbeing. The Buddhist mountains provide a good balance of scenery and settings that staff and managers can use to support and enhance tourists’ spiritual values. And governments are implementing various strategies for the devastating effects of epidemic on tourists. Guides, monks, and other professionals can be trained to address issues and promote a culture of faith, love, mercy, and other qualities in appropriate ways.

Third, this study aimed to cultivate tourist wellbeing by improving the methods applied at various Buddhist tourist sites. The model and questionnaire related to spiritual values and PWB may provide a simple way to obtain the necessary indicators. All tourists bring their own emotions, feelings, upbringing, cultural practices, and values. In the process of improving scenic destinations, we should start to consider the physical and natural environment and formulate different travel routes accordingly. Thus, we can provide programmatic support for sustainable development.

### 7.3. Limitations and future research

This study used a questionnaire to investigate the effect of tourists’ spiritual values on their PWB. This is a good basis for future research that can be integrated with other measures. Interviews and experimental methods may be used to provide additional evidence to supplement the findings. A significant limitation of this study is that it was conducted in two Buddhist mountains in China’s more developed eastern region. The findings should be tested at other religious sites to assess their generalizability. A bigger sample size could also help to support the validity of the findings.

This study analyzed the impact of spiritual values on tourists’ wellbeing in a Buddhist environment, but it is uncertain whether this effect is long-lasting. A limitation of the present study is that it merely showed correlations which could be due to other factors. Furthermore, many studies have mentioned that wellbeing affects life satisfaction. Thus, the model could be further extended to explore the interrelationship between these factors. Additionally, downstream associations between different situations, such as the moderating effects of age and religiosity, could be tested in the future. Analyzing variables that have not been previously related can lead to new discoveries and also help to reveal behavior and influences that may affect values and wellbeing. We hope this tool will help people to understand themselves better and find ways to enhance and develop themselves more fully in life.

## Data availability statement

The original contributions presented in the study are included in the article/supplementary material, further inquiries can be directed to the corresponding author.

## Ethics statement

Ethical review and approval was not required for the study on human participants in accordance with the local legislation and institutional requirements. Written informed consent from the participants was not required to participate in this study in accordance with the national legislation and the institutional requirements.

## Author contributions

GZ: conceptualization, methodology, software, investigation, formal analysis, validation, and writing—original draft. KH: conceptualization, funding acquisition, resources, validation, supervision, and writing—review and editing. SS: resources, supervision, and writing—review and editing. All authors contributed to the article and approved the submitted version.

## Funding

This research was supported by Ningbo Natural Science Foundation, National Natural Science Foundation of China (Grant No. 71904210), and Yongjiang Social Science Foundation for Yong Scholars.

## Conflict of interest

The authors declare that the research was conducted in the absence of any commercial or financial relationships that could be construed as a potential conflict of interest.

## Publisher’s note

All claims expressed in this article are solely those of the authors and do not necessarily represent those of their affiliated organizations, or those of the publisher, the editors and the reviewers. Any product that may be evaluated in this article, or claim that may be made by its manufacturer, is not guaranteed or endorsed by the publisher.
